# Redistribution of Membrane Raft Microdomains by Apolipoprotein A-I In Mouse Aortic Endothelial Cells

**DOI:** 10.47496/nl.cdm.2020.01.02

**Published:** 2020-11-13

**Authors:** Hong Yang, Ningya Zhang, Zhongmao Guo

**Affiliations:** Department of Microbiology, Immunology and Physiology, Meharry Medical College, Nashville, Tennessee, USA

**Keywords:** apolipoprotein A-I, ATP-binding cassette transport A1, caveolin-1, membrane microdomain reorganization, mouse aortic endothelial cells

## Abstract

Apolipoprotein A-I (apoAI) upregulates ATP-binding cassette transport A1 (ABCA1) in various cell types. ABCA1 has been shown to induce the redistribution of raft-associated proteins and lipids to the non-raft membrane. This report investigated the effect of apoAI on ABCA1 expression and raft cholesterol and protein distribution, as well as the effect of ABCA1 knockdown on apoAI-induced changes in mouse aortic endothelial cells (MAECs). Our data demonstrated that ABCA1 was distributed in both the lipid raft and non-raft membranes and was coimmunoprecipitated with caveolin-1 (CAV1). ApoAI treatment significantly increased the mRNA and protein levels of ABCA1 and reduced the percentage of ABCA1 in the raft membrane. Our data also showed that free cholesterol (FC) and CAV1 were concentrated in the raft-like detergent-resistant membranes (DRMs) under the control conditions. ApoAI treatment did not alter the cellular level of FC and CAV1 significantly but reduced the percentage of FC and CAV1 in the DRMs. Knockdown of ABCA1 attenuated apoAI-induced redistribution of FC and CAV1. The percentage of FC and CAV1 in the DRMs was correlated inversely with the cellular level of ABCA1, suggesting that apoAI induces relocation of CAV1 and FC from the raft to the non-rail membrane via a mechanism involving upregulation of ABCA1.

## 1. Introduction

Lipid rafts are membrane microdomains characterized by enrichment of glycosphingolipids and cholesterol [1]. Two types of lipid rafts have been documented: caveolae and non-caveolae lipid rafts. Caveolae are flask-like invaginations of the plasma membrane. The non-caveolae rafts are regions without membrane indentations and, therefore, also are referred to as planar lipid rafts [1]. ABCA1 is a membrane-associated protein and has been reported to regulate the organization of membrane microdomains. It has been reported that homozygous mutation of the ABCA1 gene increases the raft cholesterol content in fibroblasts [2]. Expression of ABCA1 in kidney cells induces the redistribution of raft lipids and proteins to the non-raft domains [3], which expands the non-raft membrane fractions and reduces the number of caveolae. It has been reported that reducing caveolae by knockout of CAV1, a caveolar structure protein, inhibited atherogenesis [4, 5], whereas re-expression of CAV1 in endothelial cells promoted atherosclerotic lesion development in mouse models [6]. However, whether ABCA1 regulates the distribution of membrane lipids and proteins among microdomains in endothelial cells has not been investigated.

Endothelial cells express considerable levels of ABCA1 [7]. Studies using rat aortic endothelial cells demonstrated that CAV1 and ABCA1 interact in the plasma membrane and co-localize in the cytoplasmic vesicles after treatment with HDLs [8, 9], and over- or down-expression of CAV1 induced increase or decrease in ABCA1 endocytosis, respectively [9]. However, research using other cell types and research approaches show conflicting results, i.e., ABCA1 was reported to localize only at the non-raft region or at both the raft and non-raft regions of the plasma membrane, dependent on the cell types and raft isolation techniques used [10–13]. ApoAI has been shown to upregulate ABCA1 expression in various cell types [14, 15]. The current report investigated the effect of apoAI on ABCA1 expression and raft cholesterol and protein distribution, as well as the effect of ABCA1 knockdown on apoAI-induced changes in MAECs.

## 2. Materials and Methods

### 2.1. siRNA knockdown of ABCA1

MAECs were obtained from Cell Biologies (Chicago, IL), and transfected with the specific siRNAs against ABCA1 or scrambled control siRNAs as described previously [16]. The knockdown efficiency was confirmed by the detection of ABCA1 proteins using Western blot analysis.

### 2.2. Detergent-Dependent Separation of Membrane Raft and Non-Raft Fractions

MAECs were incubated with 20 μg/ml apoAI or vehicle medium for 16 h and harvested in 1.1 ml of ice-cold MOPS buffer containing 1% Triton X-100 (TX100), followed by homogenizing in a Wheaton Dounce tissue grinder on ice for five strokes. The homogenate was mixed with 1.3 ml of 2.4 M sucrose solution in an SW-41 centrifuge tube and sequentially layered by 1 mL of each of the 0.8 M, 0.7 M, 0.65 M, 0.6 M, and 0.5 *M* sucrose solutions. The constructed gradient was centrifuged at 40,000 × g at 4°C for 20 h [17]. Eight fractions (1 ml/fraction) were collected from top to bottom of the gradient for the measurement of proteins and lipids.

### 2.3. Detergent-Free Separation of Membrane Raft and Non-Raft Fractions

MAECs were homogenized by passage through a 22 g × 3” needle 25 times in 1 ml of homogenization buffer containing 20 mM Tris HC1, 250 mM sucrose, 1 mM CaCl_2_, 1 mM MgCl_2_, and 10 μl/ml protease inhibitor cocktail. The homogenate was centrifuged at 1,000 × *g* for 10 min. The pellet was resuspended and homogenized as described above. The postnuclear supernatants collected following two times of homogenations were combined and mixed with an equal volume of 50% OptiPrep^™^ density gradient medium (Sigma-Aldrich, St. Louis, MO). The resulting mixture (4 ml) was placed in the bottom of a centrifuge tube. A 6 ml gradient of 0% to 20% OptiPrep^™^ medium was poured on top of the sample mixture. Gradients were centrifuged for 90 min at 52,000 × *g* using an SW-41 rotor [18] and fractionated into 1 ml fractions for measurement of proteins.

### 2.4. Immunoprecipitation

MAECs were cross-linked with 250 μM dithiobis(succinimidyl propionate) and lysed with a buffer containing 50 mM Tris HC1 (pH 8), 150 mM NaCl, 1% TX100, 2 mM EDTA and a proteinase inhibitor cocktail. The lysate was incubated sequentially with clean and CAV1 antibody-associated Pierce^™^ Protein A/G Plus Agarose beads (ThermoFisher Scientific, Waltham, MA) [19]. The agarose beads were discarded by centrifugation at 10,000 × g for 1 min. The immunoprecipitants were collected for the determination of proteins.

### 2.5. Western Blot Analysis

Proteins were resolved on 10% SDS-PAGE gels and transferred to polyvinylidene fluoride membranes. After blocking with 3% fat-free milk, the membranes were incubated with antibodies against ABCA1, CAV1, clathrin heavy chain 1 (CLTC), or β-actin (Santa Cruz Biotechnology, Santa Cruz, CA). Immunoreactive bands were visualized using chemiluminescence reagent and analysed with a GS-700 Imaging Densitometer (Bio-Rad Laboratories, Hercules, CA) [20].

### 2.6. Quantitative Real-Time Reverse Transcription (RT)-Polymerase Chain Reaction (PCR) Assay

Total RNA was extracted from MAECs using Trizol reagent (ThermoFisher Scientific), treated with DNAse I, and subjected to reverse transcription using the Applied Biosystems^™^ high-capacity cDNA reverse transcription kit (ThermoFisher Scientific). The resulting cDNAs were subjected to quantitative real-time RT-PCR with the following primers: ABCA1 forward (4’-GCTACCCACCCTACGAACAA-3), reverse (5’-GGAGTTGGATAACGGAAGCA-3’); and glyceraldehyde 3-phosphate dehydrogenase (GAPDH) forward (5’-GAGCCAAAAGGGTCATCATC-3’), reverse (5’-TAAGCAGTTGGTGGTGCAGG-3’). The expression levels of ABCA1 mRNA were normalized to GAPDH mRNA.

### 2.7. Cholesterol Analysis

Samples were mixed with a lipid extraction solvent at a sample solution-solvent ratio of 1:3 (v/v). The lipid extraction solvent contained chloroform, isopropanol, and NP-40, at a ratio of 7:11:0.1 (v/v). The phases were separated by centrifugation at 16,000 × g for 10 min. The lower lipid layer was evaporated under vacuum. The pellet was resuspended in ethanol:diethylether (3:1). Unesterified free cholesterol (FC) was measured using a cholesterol assay kit obtained from Wako Chemicals (Richmond, VA), and FC concentrations were determined based on the absorbance obtained by incubation of the cholesterol standards.

### 2.8. Statistical Analysis

Data are reported as the mean ± SEM. Differences among groups were analysed by Student’s unpaired *t*-test (for two groups) and one-way analysis of variance (for more than two groups), followed by Tukey’s post-hoc test. The correlation of the cellular ABCA1 level with the DRM CAV1 and FC levels was calculated by linear regression analysis. Statistical significance was considered as *P*<0.05.

## 3. Results

### 3.1. ApoAI Upregulated ABCA1 Expression but Did Not Alter the Interaction of ABCA1 with CAV1

**Figure 1:**
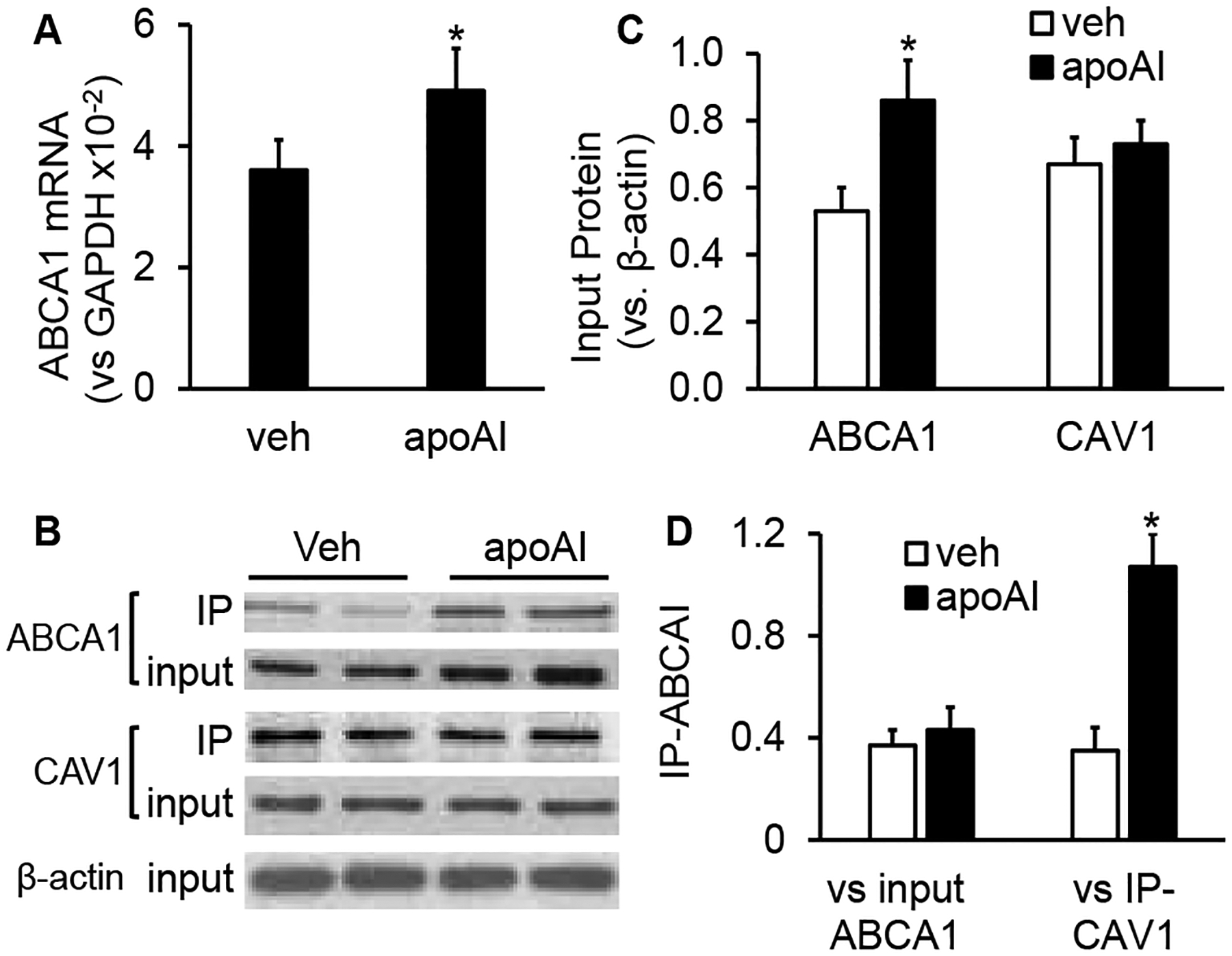
The effect of apoAI on the expression of ABCA1 and the interaction of ABCA1 and CAV1. **A**) MAECs were incubated with vehicle medium or 20μg/ml of apoAI at 37°C for 4 h. The level of ABCA1 mRNA was determined by quantitative real-time RT-PCR and expressed relative to GAPDH mRNA. **B-D)** MAECs were incubated in vehicle medium or 20μg/ml of apoAI at 37°C for 16 h. Cell lysates were subjected to immunoprecipitation with antibody against CAV1. The precipitated products and an aliquot of the cell lysate (input control) were analysed by Western blots with ABCA1 and CAV1 antibodies. The ABCA1 and CAV1 protein levels in the input control were expressed relative to b-actin (**C**). The precipitated ABCA1 was expressed relative to the immunoblot intensity of its input control or the precipitated CAV1 **(D)**. The data represent mean ± SEM of five independent experiments. * P<0.05 vs. vehicle (veh) control.

The data in (Figure 1) show that MAECs expressed ABCA1, and apoAI upregulated ABCA1 expression in these cells. Specifically, the mRNA and protein levels of ABCA1 were 36% and 62% higher, respectively, in apoAI-treated MAECs than in MAECs treated with vehicle medium (Figures 1A–1C). In contrast, apoAI treatment did not upregulate CAV1 expression, i.e., the CAV1 protein levels in MAECs treated ± apoAI were comparable (Figures 1B & 1C).

As seen in (Figures 1B & 1D), the ABCA1 protein was coimmunoprecipitated with CAV1, and apoAI increased the amount of ABCA1 coprecipitated with CAV1. Specifically, the ratio of precipitated ABCA1 relative to precipitated CAV1 (IP-ABCA1/IP-CAV1) was ~2-fold higher in MAECs treated with apoAI compared with those treated with vehicle medium (Figure 1D). However, the ratio of precipitated ABCA1 relative to the total cellular level of ABCA1 (IP-ABCA1/input ABCA1) was comparable in vehicle- and apoAI-treated cells. These results suggest that the increased amount of ABCA1 coprecipitated with CAV1 in apoAI-treated cells results from an elevated expression of ABCA1 but not an increased ability of ABCA1 to bind CAV1.

### 3.2. The Effect of ApoAI on the Distribution of ABCA1 in Membrane Microdomains

**Figure 2:**
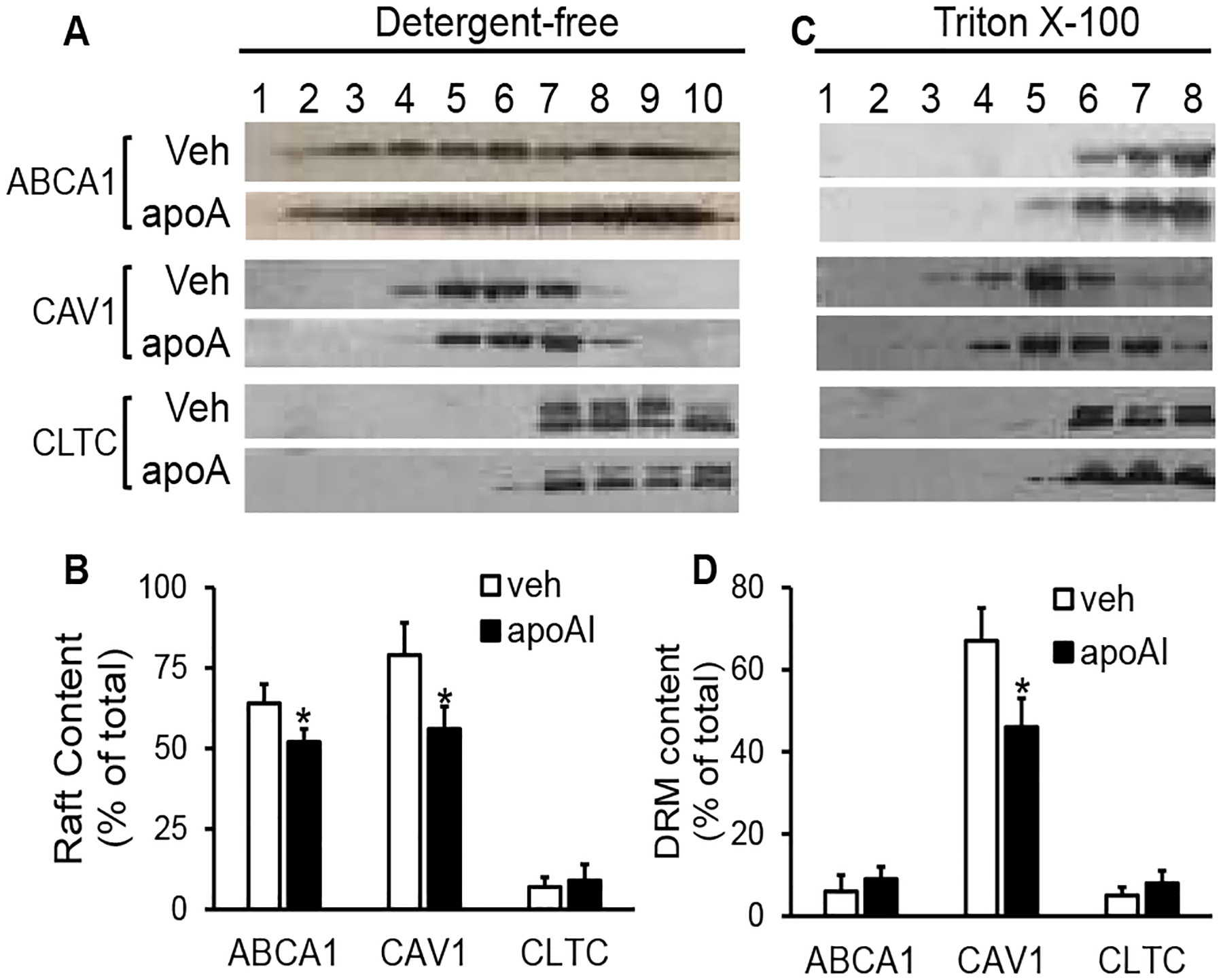
The effect of apoAI on the distribution of CAV1, ABCA1, and CLTC in membrane microdomains. MAECs were treated ± 20μg/ml of apoAI at 37°C for 16 h. **A) & B)** MAECs were homogenized by passage through a syringe needle. The postnuclear extracts were subjected to an OptiPrep^™^ gradient ultracentrifugation. The centrifuged gradients were collected into 10 fractions. **C) & D)** MAECs were homogenized in 1% cold TX100 and separated on a sucrose gradient. The centrifuged gradients were collected into eight fractions. ABCA1, CAV1, and CLTC in the gradient fractions were determined by Westem blot analysis. The percentage of proteins in the raft or raft-like DRM fractions was calculated as follows: (the amount in the raft-like DRM fractions/the total amount in all fractions) × 100. Values represent the mean ± SEM of four experiments. *P<0.05 vs. vehicle (veh) treatment.

Figure 2A illustrates typical immunoblot images of ABCA1, CAV1, and CLTC in the gradient fractions of the detergent-free lipid rail preparation. The 7^th^ to 10^th^ fractions contain CLTC, the 4^th^ to 6^th^ fractions contain CAV 1 but lack CLTC, and the 1^st^ to 3^rd^ fractions lack both CAV1 and CLTC. These three groups of gradient fractions have been considered as non-raft, caveolar, and planar raft fractions, respectively [18], Our data showed that ~64% and 52% of the ABCA1 were found in the raft fractions of the cells treated with vehicle medium or apoAI, respectively; the remaining ABCA1 exist in the non-raft fractions (Figures 2A & 2B). The difference of the raft ABCA1 percentages between vehicle- and apoAI-treated cells is statistically significant (*P*<0.05).

Isolation of DRMs has been used traditionally for studying lipid rafts [17]. It has been well-established that the raft-like DRMs migrate at ~20% sucrose, showing light-scattering bands [17]. In the current study, we observed two opaque bands in the 4^th^ to 5^th^ fractions of the TX100 detergent-dependent lipid raft preparation (data not shown). The immunoblot images in (Figure 2C) illustrate that CAV1 was distributed mainly in the 4^th^ to 5^th^ fractions of the TX100-based lipid raft preparation; thus, we designated these fractions as raft-like DRM fractions. Figure 2C also shows that ABCA1 was barely detectable in the DRM fractions and present primarily in the 6^th^ to 8^th^ fractions, coexisting with CLTC. Treatment of MAECs with apoAI did not alter the distribution of ABCA1, suggesting that ABCA1 distributes predominantly in the TX100-soluble membranes in cells treated ± apoAI.

### 3.3. The Effect of ApoAI on the Distribution of CAV1 and CLTC in Membrane Microdomains

The data in (Figures 2A & 2B) show that not all CAV1 resides in the raft membrane; a minor portion of CAV1 distributes in the non-raft domains. Specifically, ~79% of CAV1 in the vehicle-treated cells distributed in the 4^th^ to 6^th^ fractions of the detergent-free lipid raft preparation, and the remaining CAV1 distributed in the 7^th^ to 10^th^ fractions, coexisting with CLTC. In contrast, only ~56% of CAV1 was found in the 4^th^ to 6^th^ fractions in apoAI-treated cells, which was significantly less compared with that in the vehicle-treated cells (*P*<0.05).

The TX100-based technique also demonstrated apoAI-induced CAV1 relocation among membrane microdomains. Specifically, ~67% of CAV1 was located at the 4^th^ and 5^th^ fractions of the TX100-based lipid raft preparation. ApoAI treatment reduced the percentage of CAV1 in these fractions, i.e., ~46% of CAV1 was found in the 4^th^ and 5^th^ fractions in apoAI-treated cells (Figures 2B & 2C). As seen in (Figures 2C & 2D), apoAI did not alter the distribution of CLTC among membrane microdomains. CLTC existed primarily in the 6^th^ to 8^th^ fractions of the TX100-based lipid raft preparation in both apoAI- and vehicle-treated cells (Figures 2C *&* 2D).

### 3.4. The Effect ABCA1 Knockdown on ApoAI-induced Redistribution of CAV1 in Membrane Microdomains

**Figure 3:**
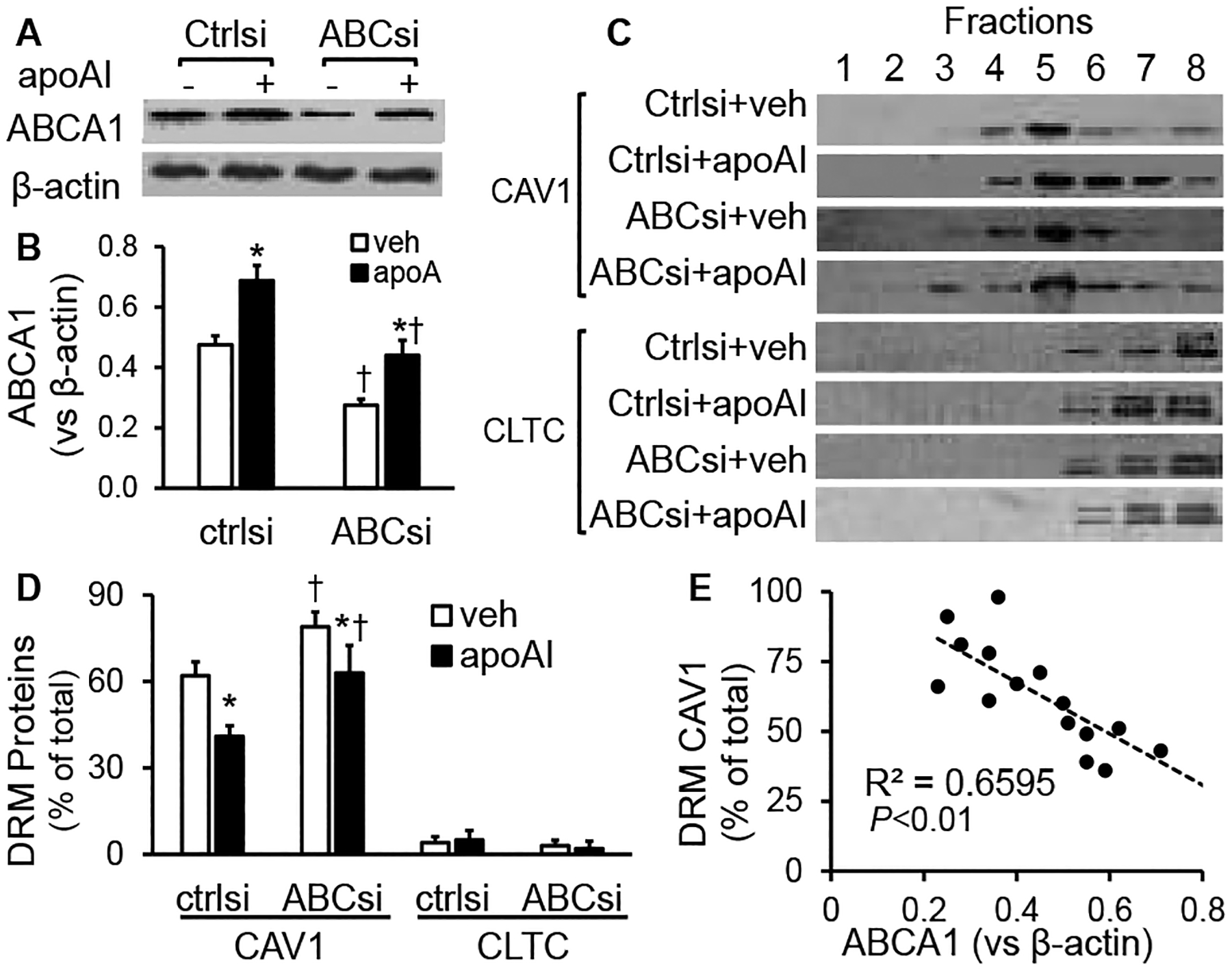
The effect of ABCA1 knockdown on apoAI-induced CAV1 redistribution in membrane microdomains. MAECs were transfected with ABCA1 siRNAs (ABCsi) or scrambled control siRNAs (ctrlsi) and then treated ± 20μg/ml of apoAI for 16 h. **A) & B)** The ABCA1 protein level was determined by Western blot analysis and expressed relative to b-actin. **C) & D)** The MAECs were homogenized in 1% cold TX100 and separated into eight fractions by sucrose density gradient centrifugation. CAV1 and CLTC in the fractions were determined by Western blot analysis. The protein level in the DRM fractions was expressed as the percentage of their total amount in all the fractions. Values represent the mean ± SEM of four experiments. *P<0.05 vs. vehicle (veh) treatment; ^†^ P<0.05 vs. cells transfected with ctrlsi. **E)** The correlation between the cellular ABCA1 protein and DRM CAV1 protein levels was calculated using the data represented in panels **B** and **C**.

Figures 3A & 3B shows that apoAI treatment upregulated ABCA1 expression in MAECs transfected with scrambled control siRNAs or ABCAI siRNAs. Transfection with ABCAI siRNAs reduced the basal and apoAI-induced ABCA1 expression.

The data in (Figures 3C & 3D) show that ~62% of CAV1 was present in the 4^th^ and 5^th^ fractions in the vehicle-treated, scrambled siRNA-transfected MAECs. Incubation of the scrambled siRNA-transfected cells with apoAI reduced the percentage of CAV1 in the DRM fractions, i.e., ~41% of CAV1 was found in the 4^th^ and 5^th^ fractions in the apoAI-treated, scrambled siRNA-transfected cells. These data were similar to those seen in the MAECs not transfected with siRNAs (Figures 2C & 2D). In contrast, siRNA-mediated ABCA1 knockdown altered the distribution of CAV1 under the vehicle or apoAI treatment conditions. As seen in (Figures 3C & 3D), ~79% and 63% of CAV1 distributed in the 4^th^ and 5^th^ fractions in the ABCA1 siRNA-transfected MAECs treated with vehicle medium or apoAI, respectively. They are significantly higher than those in the scrambled siRNA-transfected cells treated with or without apoAI, respectively. Further, siRNA knockdown of ABCA1 did not alter membrane CLTC distribution, i.e., it distributed primarily in the TX100-soluble fractions in both scrambled siRNA- and ABCA1 siRNA-transfected cells under the conditions with or without apoAI treatment (Figures 3C & 3D). Figure 3E shows that the CAV1 level in the DRM fractions was correlated inversely with the cellular level of ABCA1 (r^2^=0.6595; *P*<0.01).

### 3.5. The Effect of ApoAI Treatment and ABCA1 Knockdown on the Distribution of Cholesterol in Membrane Microdomains

**Figure 4:**
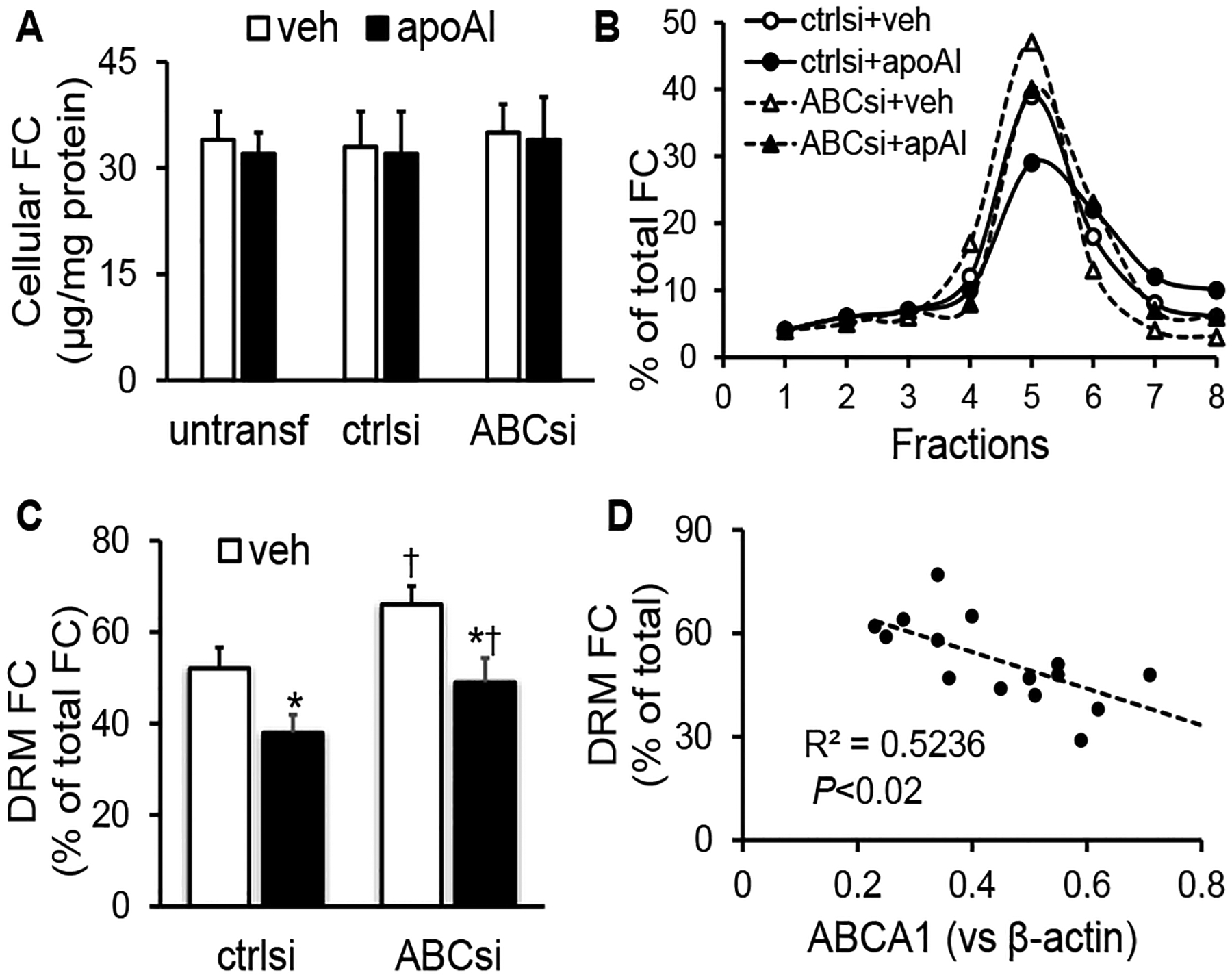
The effect of apoAI treatment and ABCA1 knockdown on cholesterol distribution in membrane domains. **A**) MAECs were transfected with ABCA1 siRNAs (ABCsi) or scrambled control siRNAs (ctrlsi), or not transfected with siRNAs (untransf). After treatment with 20μg/ml of apoAI or vehicle (veh) medium at 37°C for 16 h, cells were lysed for lipid extraction. **B) & C)** The MAECs transfected with ABCsi or ctrlsi, treated with 20μg/ml of apoAI or vehicle medium for 16 h, and then homogenized in 1% cold TX100. The homogenates were separated on a sucrose density gradient into eight fractions for lipid extraction. FC in the lipid extracts was measured enzymatically. The level of cellular FC was expressed relative to cellular proteins (μg/mg protein) (**A**). The percentage of FC in the DRMs was calculated as follows: (FC in the 4^th^ to 5^th^ fractions/total FC in all the fractions) × 100 **(C)**. Values represent the mean ± SEM of four experiments. *P<0.05 vs. veh treatment; ^†^P<0.05 vs. cells transfected with ctrlsi. **(D)** The correlation between the cellular ABCA1 protein and DRM FC levels was calculated using the data represented in (Figures 3B & 4C).

Cellular FC resides primarily in the plasma membrane, with a high concentration in the raft regions [1]. As seen in (Figure 4A), under the culture conditions without siRNA transfection, the average FC contents in MAECs treated with vehicle medium or 20 μg/ml apoAI for 16 h are ~34 μg/mg and 32 μg/mg protein, respectively. Transfection with scrambled siRNAs or ABCA1 siRNAs did not alter the FC content in vehicle- and apoAI-treated cells significantly (Figure 4A).

Figure 4B shows typical distribution curves of FC in the TX100-based lipid raft preparations of MAECs transfected with scramble siRNAs or ABCA1 siRNAs and treated with vehicle medium or apoAI. As seen in Figure 4C, apoAI treatment significantly reduced the percentage of FC in the DRMs in MAECs transfected with either scrambled siRNAs or ABCA1 siRNAs. The knockdown of ABCA1 significantly elevated the DRM FC percentage in cells treated ± apoAI. Specifically, ~52% and 38% of the FC were presented in the 4^th^ to 5^th^ fractions of the detergent-dependent raft preparations of the scrambled siRNA-transfected MAECs after treatment with vehicle medium or apoAI, respectively. In contrast, ~66% and 49% of the FC were found in the 4^th^ to 5^th^ fractions of the ABCA1 siRNA-transfected cells treated with vehicle medium or apoAI, respectively. The percentage of FC in the DRM fractions was significantly lower in cells treated with apoAI, compared with those treated with vehicle medium, and significantly higher in ABCA1 siRNA-transfected MAECs compared with scrambled siRNA-transfected cells. As seen in (Figure 4D), the percentage of FC in the DRM fractions was correlated inversely with the cellular level of ABCA1 (*r*^2^=0.5236; *P*<0.02).

## 4. Discussion

To determine the membrane microdomain distribution of ABCA1 in MAECs, the current report used two distinct methods: detergent-dependent and detergent-free lipid raft preparation. Data derived from the detergent-dependent method revealed that ABCA1 was located exclusively at the TX100-soluble fractions, consistent with previous reports showing that ABCA1 is not detectable in the TX100 DRMs [11, 12]. However, ABCA1 has been reported to reside in the cholesterol-rich membrane domains resistant to mild detergents, such as Brij 98 [13] and Lubrol WX [10]. It has been suggested that the detergent-based lipid raft separation techniques may introduce artifacts not representative of membranes [21], such as causing abnormal redistribution of glycosphingolipids and altering raft integrity. As a result, different detergents may yield varying subsets of DRMs, each with unique properties [21].

To avoid the complications associated with detergent extraction procedures, the current report examined the membrane distribution of ABCA1 using a detergent-free lipid raft preparation method. Our data suggest that ABCA1 was distributed in both the raft and non-raft membranes. In the raft membranes, ABCA1 either coexisted with CAV1 or distributed in the domains lacking both CLTC and CAV1. Namely, ABCA1 resides in both the caveolar and planar raft membranes. Our co-immunoprecipitation study also demonstrated that ABCA1 could be precipitated along with CAV1, which provided evidence for the interaction of *ABCA1* with CAV1. In addition, a previous report demonstrated the presence of ABCA1 in the membrane domains resistant to Lubrol WX, along with flotillin-1, a marker protein of planar raft [10]. Taken together, findings from the current study and previous reports suggest that at least a portion of ABCA1 is located at the raft membrane regions, which are soluble in TX100. Further, it has been suggested that ABCA1 resides in a border region between the raft and non-raft domains [22].

Although it is not clear why detergents alter the membrane distribution of some proteins and lipids (e.g., ABCA1 and glycosphingolipids) but do not affect others (e.g., caveolin and cholesterol), detergent-based methods still are invaluable tools for studying cellular rafts and characterizing their composition [17]. The insolubility of rafts in nonionic detergents has enabled the isolation of DRMs, which, like rafts, float away from detergent-soluble proteins and lipids. Data from the current report provides evidence that FC was concentrated in the raft-like DRM fractions, and apoAI redistributed FC from the DRM to the detergent-soluble fractions. In addition, our data demonstrated that CAV1 distributed primarily in the DRM fractions and treatment of MAECs with apoAI induced relocation of CAV1 from the DRM to the detergent-soluble fractions. These results agree with the data derived from the detergent-free method. The membrane microdomain reorganization induced by apoAI in endothelial cells may be important physiologically, as the proteins and lipids targeted in lipid rafts have been implicated in many endothelial cell activities, such as transport of macromolecules across the endothelium [4], regulation of the tonic vasoconstriction [23], and oxidative stress responses [24].

Other important findings from the current report are that apoAI upregulated ABCA1 expression, knockdown of ABCA1 attenuated apoAI-induced relocation of CAV1 and raft cholesterol, and the percentage of CAV1 and cholesterol in the raft-like DRM is related negatively to the level of total ABCA1 in MAECs. These findings suggest that the upregulation of ABCA1 expression is a mechanism for apoAI-induced CAV1 and raft cholesterol relocation to the non-raft domains. The mechanism for ABCA1-induced membrane microdomain reorganization remains poorly understood [11]. The best-known activity of ABCA1 is to convey cholesterol and phospholipids between the Golgi and cell membrane and transport them to cell surface-bound apoAI [25]. It has been postulated that the active lipid translocation via ABCA1 and the flop of phospholipids and FC from the inner to the outer leaflet of the plasma membrane induces membrane destabilization, leading to a shift of microdomain lipids and proteins [11].

## 5. Conclusion

Our data suggest that ABCA1 distributes in both the lipid raft and non-raft membranes in MAECs. ApoAI upregulates ABCA1 expression and induces the CAV1 and raft cholesterol shift to the non-raft membrane. The knockdown of ABCA1 attenuates apoAI-induced relocation of and raft cholesterol. These findings add to the growing repertoire supporting the role of ABCA1 in the organization of membrane microdomains; the higher the ABCA1 expression in cells, the less the raft domains occur on the plasma membrane.
